# Laparoscopic Repair of a Left Retrocaval Ureter in a 16-Year-Old Girl

**DOI:** 10.1055/s-0039-1678567

**Published:** 2019-02-22

**Authors:** Anna Kadar, Lorena Vatra, Anca Avram, Daniela Stoica, Marcel Oancea

**Affiliations:** 1 Department of Pediatric Surgery, Clinical Emergency Hospital for Children “Marie Curie,” Bucharest, Romania; 2 Department of Pediatric Radiology, Clinical Emergency Hospital for Children “Marie Curie,” Bucharest, Romania

**Keywords:** left retrocaval ureter, situs inversus, fish hook sign, laparoscopic ureteroureterostomy, left circumcaval ureter

## Abstract

Left retrocaval ureter is an extremely rare congenital malformation which is associated with situs inversus, duplicated or translated inferior vena cava (IVC). We report a female adolescent who presented with a history of intermittent, colicky lumbar pain. Diagnostic workup revealed left retrocaval ureter and left ureterohydronephrosis. The girl underwent laparoscopy. The renal pelvis and ureter posterior to the vena cava were dissected, transected at the caudal point of the dilated ureteral segment, and uncrossed and repositioned lateral to the vena cava. Ureteroureterostomy was performed over a double-J ureteral stent after spatulation of the distal ureter. The postoperative course was uneventful and the ureteral stent removed after 5 weeks. During follow-up, the patient is symptom-free. Our case demonstrates that our laparoscopic approach is feasible in this rare anatomic anomaly.

## Introduction

Retrocaval ureter is a rare congenital anomaly where the ureter passes posterior to the inferior vena cava (IVC) resulting in extrinsic obstruction and corresponding progressive ureterohydronephrosis. 1 It is usually encountered on the right side. Left retrocaval ureter is an extremely rare condition, and is associated with situs inversus, duplicated or single left IVC. The treatment of choice is surgery. Laparoscopy offers good results. 2

We report a case of a left retrocaval ureter with consequent ureterohydronephrosis in an adolescent girl with situs inversus abdominalis. A laparoscopic transperitoneal approach was used for correction of the anomaly.

## Case Report

A 16-year-old girl presented with a history of intermittent colicky left lumbar pain. The acute episodes of pain were related to excessive hydration, physical activity, or diuretic drinks. There was no history of urinary tract infection or hematuria. Laboratory urinary tests revealed normal values. Ultrasonography showed dilatation of the left renal pelvis and proximal ureter, with normal renal parenchymal thickness and no calculus; an abdominal situs inversus.

**Fig. 1:**
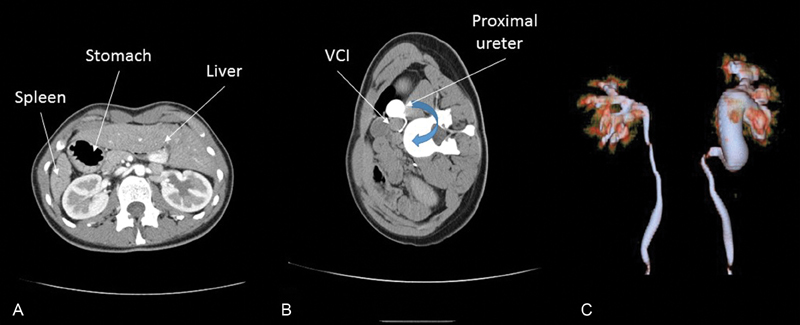
Abdominal computed tomography scan with contrast injection. ( **A** ) Axial view, dorsal decubitus position: abdominal situs inversus. ( **B** ) Axial view, lateral decubitus position: proximal dilated ureter with retrocaval path. ( **C** ) 3D reconstruction: “hook-fish” sign. VCI, vena cava inferior.

A computed tomography confirmed the abdominal situs inversus, and described left ureterohydronephrosis, and left retrocaval ureter. The anteroposterior renal pelvis diameter (APD) was 20 mm and the proximal ureter had a diameter of 18 mm; parenchymal index was normal ( Fig. 1 ).

As the patient was symptomatic, she was taken into surgery. We used the laparoscopic transperitoneal approach.

**Fig. 2:**
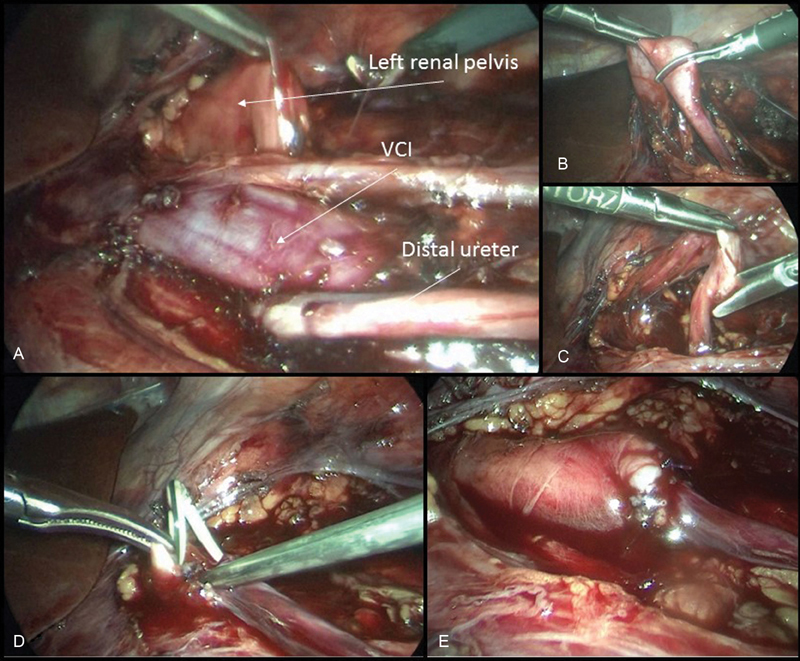
Intraoperative images. ( **A** ) Retrocaval disposition of the left ureter. ( **B** , **C** ) Transection and spatulation of the distal ureter. ( **D** ) Intraoperative placement of a J–J ureteral stent. ( **E** ) Final aspect of the anastomosis. VCI, vena cava inferior.

After induction of general endotracheal anesthesia, a nasogastric tube and a Foley's catheter were inserted. The patient was placed in a modified flank position (45 degrees right lateral decubitus position) with overextension. A 5 mm umbilical port was placed for the 30-degree laparoscope, using an open technique. Two additional 5 mm ports were carefully placed. The line of Toldt's was incised and the left colon was retracted medially to permit access to the renal and retroperitoneal space. Dissection was carried using blunt and sharp instruments, exposing the renal pelvis, precaval dilated ureter, preureteral segment of the IVC, and postcaval ureter. After mobilizing the retrocaval ureteral segment, no intrinsic stricture was noted. The ureter was transected at the most caudal point of the dilated segment, mobilized, uncrossed, and placed lateral to IVC. After spatulation of the distal ureter and antegrade placement double-J ureteral stent (Charrière 4.7) we performed a ureteroureterostomy over a using a 5–0 monofilament, resorbable suture ( Fig. 2 ). An intraperitoneal drain was placed close to the anastomosis. Operative time was 150 minutes; no significant blood loss was noted.

The postoperative course was uneventful; the lumbar drain was removed on the 2nd postoperative day. The girl was discharged home after 5 days. The double-J stent was removed after 5 weeks. During a 20-month follow-up the patient was symptom-free. Ultrasonographic re-evaluation showed marked decrease of APD (7 mm).

## Discussion

Retrocaval ureter, also known as circumcaval ureter or preureteral vena cava, is a rare congenital anomaly due to the abnormal development of the inferior vena cava. The embryological event causing the malformation is the persistence of the right posterior cardinal vein instead of the right subcardinal vein as the renal segment of the cava vein. 1 The term preureteral vena cava is preferred to retrocaval ureter, as the anomaly generating this condition is a vascular one.

It was first described by Hochstetter in 1893. 3 Its incidence is 1 in 1,500 cadavers, and the sex ratio is 3:1, male to female. The first reported ureteropyeloplasty was performed by Anderson and Hynes in 1949, in a 10–year- old girl, with this anomaly. 4

Based on imaging, two types of retrocaval ureter have been described. The radiological classification of Bateson and Atkinson distinguishes type I–obstructive–“low loop” (ureter passes behind the IVC at the level of 3rd lumbar vertebra, with a fish-hook [S–shape] appearance), and type II–nonobstructive–“high loop” (retrocaval segment is at the level or the renal pelvis). 5 Symptoms usually develop in the third or fourth decade of life, children are less often diagnosed with this condition. There are also asymptomatic forms which do not require surgical treatment; type II, nonobstructive cases (approximately 10%). 5

If the subcardinal vein persists at a left vena cava system, left circumcaval ureter is encountered. It is an exceptionally rare anomaly and associated with either complete or partial abdominal situs inversus, duplicated IVC or single left IVC. There are only nine cases of left retrocaval ureter published in the medical literature 6 7 8 9 10 11 12 13 14 ( Table 1 ). Among them, there is only one pediatric case: a 10-month-old girl with Goldenhar's syndrome, diagnosed during routine urological screening. 14

**Table 1:** Published cases of left retrocaval ureter

No.	Author	Patient's age	Associated anomaly
1	Brooks, 1962 6	43 y	Total situs inversus
2	Pierro, 1990 7	34 y	Left IVC transposition
3	Watanabe, 1991 8	68 y	Partial situs inversus
4	Ishitoya, 1997 14	10 mo	Left IVC transposition
5	Rubinstein, 1999 9	45 y	Duplicated IVC
6	Gramegna, 2003 10	19 y	Left IVC transposition
7	Kozyrakis, 2012 11	75 y	Left IVC transposition
8	Kim, 2013 12	24 y	Duplicated IVC
9	Thirugnanasambandam, 2015 13	84 y	Left IVC transposition

Abbreviation: IVA, inferior vena cava.

The treatment includes ureteral division, relocation, and ureteroureterostomy with or without ureteral segmental resection. It can be performed as open surgery or using minimal invasive techniques. Laparoscopic transperitoneal treatment for this condition was first described in adults by Baba et al in 1994 15 and retroperitoneal approach was first used in 1999. 16 17 In children, laparoscopic approach has been used in 2002. 18 The first case of robot-assisted laparoscopic correction of a retrocaval ureter was done in a child in 2006. 19 Resection of the retrocaval segment is described and may be needed, due to fibrosis, dysplasia, or narrowing of the retrocaval ureteral segment. We did not perform any ureteral resection, as we did not encounter any intrinsic obstruction. Spatulation of the distal ureter allowed us to perform a wide anastomosis, using separate stitches, over an ureteral stent. Ureteroureterostomy was the most challenging and time-consuming part of the intervention. Running-sutures may provide a faster intracorporeal suture. Transperitoneal intracorporeal suturing is less time-consuming and easier than retroperitoneal suturing. 20 A minilaparotomy to perform the anastomosis, after finishing the laparoscopic dissection and transection of the ureter, can be used to reduce operative time. 21

## Conclusions

There is no consensus or surgical gold standard to treat this malformation. The laparoscopic transperitoneal approach to treat retrocaval ureter is a feasible technique with an excellent postoperative result in children.

## Notes

**New Insights and the Importance for the Pediatric Surgeon**
Retrocaval ureter is a rare malformation that can be efficiently treated by laparoscopic surgery. Left retrocaval ureter is exceptionally rare and is associated with caval vein anomaly.
